# Diversity in social networks and brain aging in community-dwelling older adults in Japan: The NEIGE Study

**DOI:** 10.1093/geroni/igaf122.2963

**Published:** 2025-12-31

**Authors:** Hiroshi Murayama, Shiho Amagasa, Masaki Machida, Shigeru Inoue, Takeo Fujiwara, Yugo Shobugawa

**Affiliations:** Tokyo Metropolitan Institute for Geriatrics and Gerontology, Tokyo, Japan; Teikyo University, Tokyo, Tokyo, Japan; Tokyo Medical University, Tokyo Medical University, Tokyo, Japan; Tokyo Medical University, Tokyo Medical University, Tokyo, Japan; Institute of Science Tokyo, Tokyo, Tokyo, Japan; Niigata University, Niigata, Niigata, Japan

## Abstract

As the global population ages, understanding factors influencing brain health in older adults is crucial due to its impact on cognitive decline. Age-related changes in key regions like the hippocampus, gray matter, and prefrontal cortex are associated with cognitive decline. This study investigated the influence of social network structure on brain aging in older Japanese adults, focusing on regions linked to cognitive decline. We used longitudinal data from the Neuron to Environmental Impact across Generations Study (NEIGE Study), which is a prospective cohort study of randomly-sampled community-dwelling individuals aged 65–84 years living in Tokamachi City, Niigata Prefecture, Japan. The baseline survey was conducted in 2017 and included 527 independent older people. The follow-up survey was completed in 2021. This study analyzed 279 who had magnetic resonance imaging at both baseline and follow-up (47.6% men; mean age: 73.0 ± 5.1 years). Social network homogeneity/heterogeneity was assessed based on similarities or diversity in relationships, and factor analysis identified two network types. Multiple linear regression analyses with inverse probability weighting indicated that homogeneous social networks were not associated with brain volume changes. However, lower heterogeneity in social networks was linked to greater hippocampal volume decline, after adjusting for sociodemographic factors and health behaviors. This association was prominent in right hippocampus. No significant associations were observed for gray matter or prefrontal cortex. More heterogeneous social networks may help mitigate age-related decline in hippocampal volume in older adults, suggesting the cognitive health benefits of social diversity. Further research is needed to explore underlying mechanisms.

